# Non-canonical binding interactions of the RNA recognition motif (RRM) domains of P34 protein modulate binding within the 5S ribonucleoprotein particle (5S RNP)

**DOI:** 10.1371/journal.pone.0177890

**Published:** 2017-05-18

**Authors:** Anyango D. Kamina, Noreen Williams

**Affiliations:** Department of Microbiology and Immunology and Witebsky Center for Microbial Pathogenesis and Immunology, University at Buffalo, Buffalo, New York, United States of America; University of Edinburgh, UNITED KINGDOM

## Abstract

RNA binding proteins are involved in many aspects of RNA metabolism. In *Trypanosoma brucei*, our laboratory has identified two trypanosome-specific RNA binding proteins P34 and P37 that are involved in the maturation of the 60S subunit during ribosome biogenesis. These proteins are part of the *T*. *brucei* 5S ribonucleoprotein particle (5S RNP) and P34 binds to 5S ribosomal RNA (rRNA) and ribosomal protein L5 through its N-terminus and its RNA recognition motif (RRM) domains. We generated truncated P34 proteins to determine these domains’ interactions with 5S rRNA and L5. Our analyses demonstrate that RRM1 of P34 mediates the majority of binding with 5S rRNA and the N-terminus together with RRM1 contribute the most to binding with L5. We determined that the consensus ribonucleoprotein (RNP) 1 and 2 sequences, characteristic of canonical RRM domains, are not fully conserved in the RRM domains of P34. However, the aromatic amino acids previously described to mediate base stacking interactions with their RNA target are conserved in both of the RRM domains of P34. Surprisingly, mutation of these aromatic residues did not disrupt but instead enhanced 5S rRNA binding. However, we identified four arginine residues located in RRM1 of P34 that strongly impact L5 binding. These mutational analyses of P34 suggest that the binding site for 5S rRNA and L5 are near each other and specific residues within P34 regulate the formation of the 5S RNP. These studies show the unique way that the domains of P34 mediate binding with the *T*. *brucei* 5S RNP.

## Introduction

Ribosome biogenesis is a conserved cellular process that is essential in all organisms. This process requires the coordination of ribosomal RNAs (rRNA), ribosomal proteins and over 200 accessory factors to form functional ribosomes [1, 2]. In eukaryotes, ribosome biogenesis begins in the nucleolus where three of the four rRNA species are co-transcribed as a 35S precursor rRNA [3]. The fourth rRNA, 5S rRNA is transcribed in the nucleoplasm (except in *Saccharomyces cerevesiae* where this process takes place in the nucleolus) [4]. 5S rRNA then forms a nucleoplasmic 5S ribonucleoprotein particle (5S RNP) with ribosomal protein L5 and is trafficked into the nucleolus [5]. Once in the nucleolus, additional components join the complex including ribosomal protein L11 and the assembly factors Rpf2 and Rrs1, which function to properly assemble the other members of the 5S RNP to the 60S pre-ribosomal subunit [5–9]. The 35S precursor rRNA is subsequently cleaved and processed into rRNA components that together with ribosomal and non-ribosomal proteins form the 43S and 66S pre-ribosomal subunits [10]. These two subunits are further modified and are exported to the cytoplasm as 60S and pre-40S pre-ribosomal subunits. The pre-40S undergoes its final maturation steps and joins with the mature 60S to form the 80S ribosome that performs protein synthesis [10].

Although many aspects of ribosome biogenesis are conserved in eukaryotes, several unique components have been identified in the parasite *Trypanosoma brucei*, the causative agent of African trypanosomiasis in humans and livestock [11–13]. One major difference in *T*. *brucei* ribosome biogenesis identified by our laboratory is the involvement of two trypanosome-specific RNA binding proteins, P34 and P37 in this pathway [14, 15]. These two proteins are involved in the maturation of the 60S pre-ribosomal subunit and also assemble in the nucleoplasmic and nucleolar 5S RNP [16–18]. Characterization of these two proteins suggests that they have similar functional and biochemical properties. Structurally, both P34 and P37 contain two central RNA recognition motifs (RRM). In the C-terminal KKDX repeat domain, they have a putative nuclear export signal as well as putative nucleolar and nuclear localization signals. The major difference between P34 and P37 is the presence of 18 extra amino acids in the N-terminal region of P37. Knockdown of these two proteins by RNA interference leads to a loss of cell viability, a decrease in 5S rRNA levels, and defects in ribosome formation [14]. *In vitro* studies of the interactions within the *T*. *brucei* nucleoplasmic 5S RNP showed that specifically the N- terminus and the RRM domains of P34 are involved in mediating interactions with ribosomal protein L5 and with 5S rRNA [19, 20]. Because these proteins have been shown to be structurally and functionally similar, we characterized the P34 protein in these studies.

RRMs are RNA binding domains that have been shown to mediate RNA binding and in some instances also mediate protein binding [21]. RRM domains are canonically composed of four β-strands packed against two α-helices that fold into a βαββαβ topology. These domains recognize their RNA target through consensus sequences ribonucleoprotein (RNP) 1 and 2 that are located in the β1 and β3 strands [21, 22]. Although these consensus sequences are involved in binding to the RNA target, studies have shown that in some instances, the elements flanking the RRM domains (the N-and/or C-terminus) and/or the linker regions (between the folds of the RRM domains or the ones joining the RRM domains) confer specificity of binding [23]. The ability of the RRM domains of P34 to mediate binding to both 5S rRNA and L5 protein led us to further investigate how these domains of P34 function. First, we evaluated how the RRM domains and flanking regions of P34 interact with 5S rRNA and L5 protein. Second, we identified the residues in the RRM domains of P34 that are involved in mediating interactions between 5S rRNA and L5. From these studies, we have provided another example of how RRM domains use versatile mechanisms to mediate not only protein-RNA interactions but also protein-protein interactions.

## Materials and methods

### Recombinant proteins

**Fig 1:**
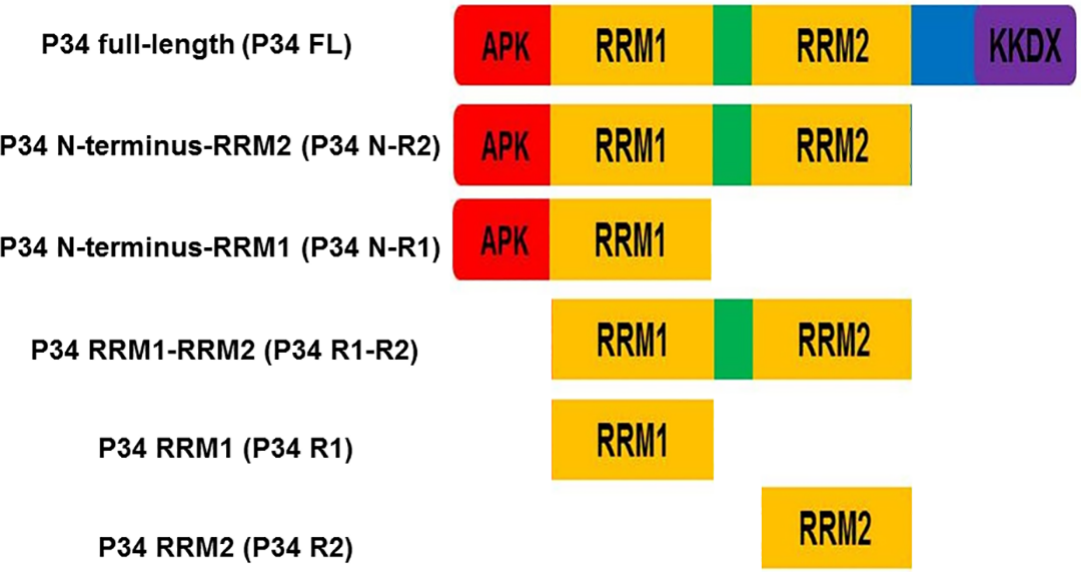
P34 protein truncates used in this study. Schematic diagram of P34 protein truncates.

P34 and L5 were individually cloned into the pTrcHis TOPO vector (Thermo Fisher Scientific) and expressed as N-terminal poly-(His)_6_ tagged proteins in TOP10 One Shot *E*. *coli* cells (Thermo Fisher Scientific). Specific primers were designed (Table 1) and used to amplify different regions of P34 in order to generate truncates of the P34 protein (Fig 1) and used in site-directed mutagenesis (GeneArt, site-directed mutagenesis system, Thermo Fisher Scientific) to generate mutated P34 proteins.

**Table 1:** List of the oligonucleotides used in this study.

Primer used in this study	Sequence (5’-3’)
P34 F	ATG GCC CCA AAG TCT GCT GC
P34 R	TCA CTG CTT CCT CTT GGC AT
P34 N-terminus-RRM2 F	ATG GCC CCA AAG TCT GCT GC
P34 N-terminus-RRM2 R	TCA GCG AGT TGA AAG AGC CAC ACG TAG
P34 N-terminus-RRM1 F	ATG GCC CCA AAG TCT GCT GC
P34 N-terminus-RRM1 R	TCA GGT TTT GGC AGG GCT TAC GGT CAC
P34 RRM1-RRM2 F	AAC GGT GTG TAT GTG AAG AAC TGG
P34 RRM1-RRM2 R	TCA GCG AGT TGA AAG AGC CAC ACG TAG
P34 RRM1 F	AAC GGT GTG TAT GTG AAG AAC TGG
P34 RRM1 R	TCA GGT TTT GGC AGG GCT TAC GGT CAC
P34 RRM2 F	TCC GTT GTG TTC CTG TCG CCC ATC
P34 RRM2 R	TCA GCG AGT TGA AAG AGC CAC ACG TAG
P34 Y60A F	GGC ACC CAC AAC GGT GTG GCA GTG AAG AAC TGG GGT CAG
P34 Y60A R	CTG ACC CCA GTT CTT CAC TGC CAC ACC GTT GAG GGT GCC
P34 Y92A F	CAG ATC CGT CGG CGC CGC GCC ATT ATC TTC TTC GAG
P34 Y92A R	CTC GAA GAA GAT AAT GGC GGC GCG GCG CCG ACG GAT CTG
P34 F141A F	GAG GGC TCT TCC GTT GTG GCC CTG TCG CCC ATC TTC CGT
P34 F141A R	ACG GAA GAT GGG CGA CAG GGC CAC AAC GGA AGA GCC CTC
P34 Y174A F	CGG ACG TAC CAT CAG AAC GCT GCT TAC GTG TAC CTT GAC
P34 Y174A R	GTC AAG GTA CAC GTA AGC AGC GTT CTG ATG GTA CGT CCG
P34 Y176A F	TAC CAT CAG AAC TAT GCT GCC GTG TAC CTT GAC TCG GCT
P34 Y176A R	AGC CGA GTC AAG GTA CAC GGC AGC ATA GTT CTG ATG GTA
P34 R88-91A F	AGT GCT CAG ATC GCC GCA GCG GCC TAC GCC ATT ATC
P34 R88-91A R	GAT AAT GGC GTA GGC CGC TGC GGC GAT CTG AGC ACT
P34 R88-91K F	GTC AGT GCT CAG ATC AAA AAG AAG AAA TAC GCC ATT ATC TTC
P34 R88-91K R	GAA GAT AAT GGC GTA TTT CTT TTT CTT GAT CTG AGC ACT GAC
P34 R91A, Y92A F	CAG ATC CGT CGG CGC GCA GCG GCA ATT ATC TTC TTC GAG
P34 R91A, Y92A R	CTC GAA GAA GAT AAT TGC CGC TGC GCG CCG ACG GAT CTG
5ST3 Fwd	ATTAACCCTCACTAAAGGGTACGACCATACTTGGCC [17]
5S Rev	AGAGTACAACACCCCGGGT [17]

DNA sequence analysis was used to confirm that all the recombinant protein expression plasmids were properly cloned. These P34 truncates and mutated P34 proteins were expressed from the pTrcHis TOPO vector (Thermo Fisher Scientific) as described below. Expression of recombinant proteins was induced with 1 mM IPTG for 5 hours at 37°C. Cell pellets were frozen at -80°C, thawed, resuspended in 1X native purification buffer (50 mM NaH_2_PO_4_ pH 8.0, 500 mM NaCl) and incubated with lysozyme (final concentration 1mg/ml) for 1 hour at 4°C. Recombinant proteins were then purified as previously described [16]. Eluted fractions of the recombinant proteins were analyzed by SDS polyacrylamide gel electrophoresis and visualized with Coomassie blue staining and by western blotting (S1 Fig). Fractions containing the recombinant proteins were aliquoted as 200 μl fractions and frozen at -80°C.

### Filter binding assays

5S rDNA (cloned into a pCR TOPO 2.1 vector, Thermo Fisher Scientific) was amplified using a forward primer containing a T3 promoter sequence and a reverse primer (Table 1) to generate a template that was used in a T3-driven *in vitro* transcription of radiolabeled [α-^32^P] UTP 5S rRNA (MaxiScript, Thermo Fisher Scientific). The radiolabeled 5S rRNA was separated from unincorporated nucleotides using Nuc-Away spin columns (Thermo Fisher Scientific). For the filter-binding assays, the radiolabeled 5S rRNA was kept at a constant concentration (0.05 nM) and incubated with increasing concentrations (0–300 nM) of recombinant proteins (full length, truncated, or mutated P34 proteins). The protein-RNA mixture was incubated in binding buffer (10 mM Tris pH 7.4, 1 mM EDTA, 100 mM NaCl and 100 μg/ml BSA) for 30 minutes at room temperature. After incubation the reactions were applied to a nitrocellulose membrane (to capture protein bound 5S rRNA) underlaid with a nytran membrane (to capture the unbound 5S rRNA), and then filtered by vacuum (Bio-dot vacuum filtration apparatus, Bio-Rad). The radioactivity associated with both membranes was measured using a phosphor-imager (Bio-Rad). The amount of bound rRNA for each protein concentration used was calculated as a ratio of the signal obtained from the nitrocellulose membrane to the total signal on both membranes (percent 5S rRNA bound = [bound/(bound + unbound)]. The binding affinities (Kd) of 5S rRNA for the recombinant P34 proteins were calculated using GraphPad prism 5 using the equation Y = Bmax*(X/Kd +X). Y = bound 5S rRNA, Bmax = 100%, the theoretical maximal signal that can be obtained if all of the RNA is bound to the protein in the reaction mixture, X = concentration of protein Kd = binding affinity. The filter binding figures are an average of biological triplicates. Representative images of the nitrocellulose and nytran membranes are shown (S2 Fig).

### Immune capture experiments

The preparation of antibody-coated beads was performed as previously described [20]. The beads were coated with anti-P34/P37 antibody [15] and incubated with recombinant proteins (1 μg each) for 1 hr at 4°C with gentle rotation. After incubation, the supernatants were removed and the beads, coated with antibody-protein complexes, were washed three times in PBS-T (PBS-tween 20). The protein complexes were eluted from the beads by resuspending them in SDS sample buffer and then heating at 70°C for 10 minutes. The eluted fractions (bound L5), together with the supernatant (unbound L5) and the input (antibody control), were analyzed by SDS polyacrylamide gel electrophoresis followed by western blotting using anti-L5 antibody [16]. To determine the amount of L5 protein bound to the P34 truncates or mutants,we quantified the signal associated with the pellet (P) fractions (representing the bound L5) in each reaction. We then normalized the values obtained from these quantifications to the values obtained from the wild type reaction (L5 + P34 FL), which was set to 1. These experiments were performed with at least three biological replicates. Representative blots are shown and the quantified mean with the calculated standard error of the mean (SEM) are shown as bar graphs underneath the blots.

## Results

### The RRM1 domain of P34 mediates most of the binding between P34 and 5S rRNA

**Fig 2:**
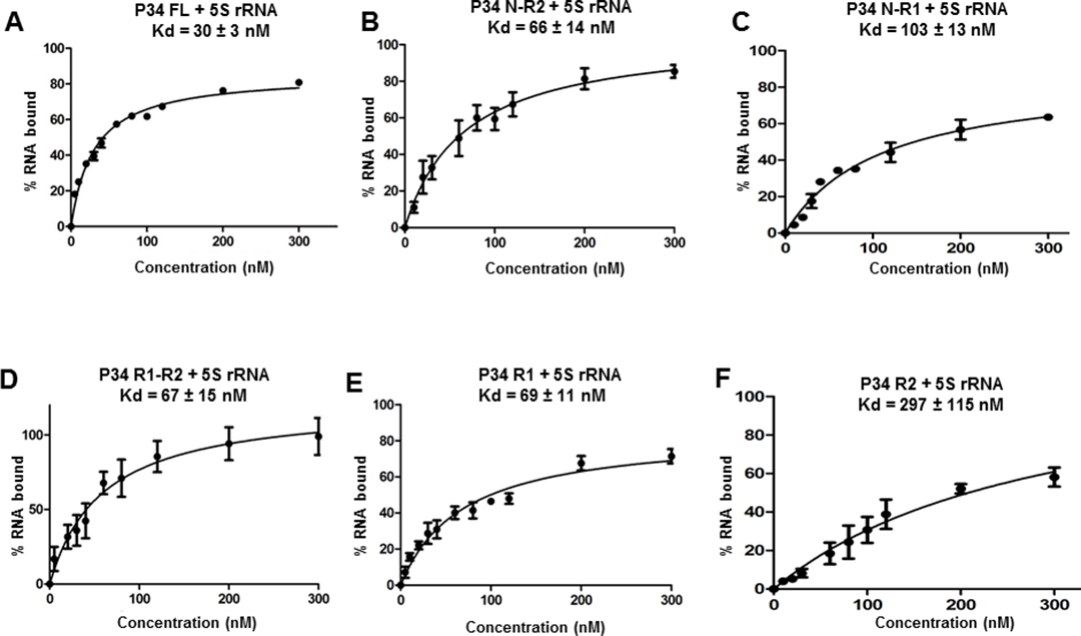
RRM1 domain of P34 contributes the most in mediating binding between P34 and 5S rRNA. Recombinant proteins were incubated with radiolabeled 5S rRNA and their interactions were analyzed using filter binding assays. The values obtained from the quantification of the bound and the free 5S rRNA (bound/[bound +free]) were used to calculate the binding affinity (Kd) of 5S rRNA for the P34 full-length protein (P34 FL, positive control) and for the truncated P34 proteins.

Previous studies from our laboratory showed that the RRM domains and the N-terminal domain of P34 are important in mediating interactions with 5S rRNA and that the C-terminal domain alone did not bind to 5S rRNA [19]. Based on these studies, we generated truncated P34 proteins that consisted of the N-terminus with the RRM domains, the RRM domains together, and the RRM domains individually in order to further characterize how these domains of P34 mediate binding with 5S rRNA (Fig 1). These recombinant protein truncates were prepared in *E*. *coli* and their interaction with 5S rRNA was characterized using filter binding assays. From these experiments, the calculated binding affinity of 5S rRNA for the full length P34 protein under our conditions was 30 ± 3 nM (Fig 2. Panel A). In the absence of the C-terminus, the binding affinity of 5S rRNA for P34 decreased two fold (Kd = 66 ± 14 nM) compared to the binding affinity of 5S rRNA for the full-length P34 (Fig 2. Panel B). In the absence of both the RRM2 and the C-terminus domains of P34, the binding affinity decreased three fold (Kd = 103 ± 13 nM) relative to the binding affinity for the full-length P34 protein (Fig 2. Panel C). These results show that the RRM2 domain and the C-terminus of P34 contribute to the binding affinity of 5S rRNA for P34.

The binding affinity of 5S rRNA for the RRM domains alone (Fig 2. Panel D) was two fold less (Kd = 67 ± 15 nM) than the binding affinity of 5S rRNA for the full-length P34 protein (Fig 2. Panel A). This result is similar to the calculated binding affinity of 5S rRNA for the P34 construct lacking only the C-terminus (Fig 2. Panel B). Comparing this result to the binding affinity of 5S rRNA for the P34 construct lacking both the C-terminus and RRM2 (Fig 2. Panel C) and the construct lacking the C-terminus alone (Fig 2. Panel B), we hypothesized that the region of P34 that contributes the most to binding with 5S rRNA is located in the RRM domains. To test this hypothesis, we performed filter binding assays between 5S rRNA and each of the P34 RRM domains individually. Results from these experiments showed that RRM1 (Fig 2. Panel E) or RRM2 (Fig 2. Panel F) alone have a binding affinity to 5S rRNA that decreased two fold (Kd = 69 ± 11 nM) and tenfold (Kd = 297 ± 115 nM) respectively relative to full-length P34 protein (Fig 2. Panel A). Therefore, we concluded that the RRM1 domain of P34 mediates majority of the binding between P34 and 5S rRNA (compare Fig 2. Panels B-E). Interestingly, when the N-terminus is combined with RRM1 alone there was a small negative effect on 5S rRNA binding but this was not observed when the N-terminus was combined with both RRM1 and RRM2 (compare Fig 2. Panel B and Panel C). This result suggests that one of the roles of RRM2 could be to alleviate this negative effect on 5S rRNA binding.

### The N-terminus and RRM1 of P34 mediate most of the binding between P34 and L5

**Fig 3:**
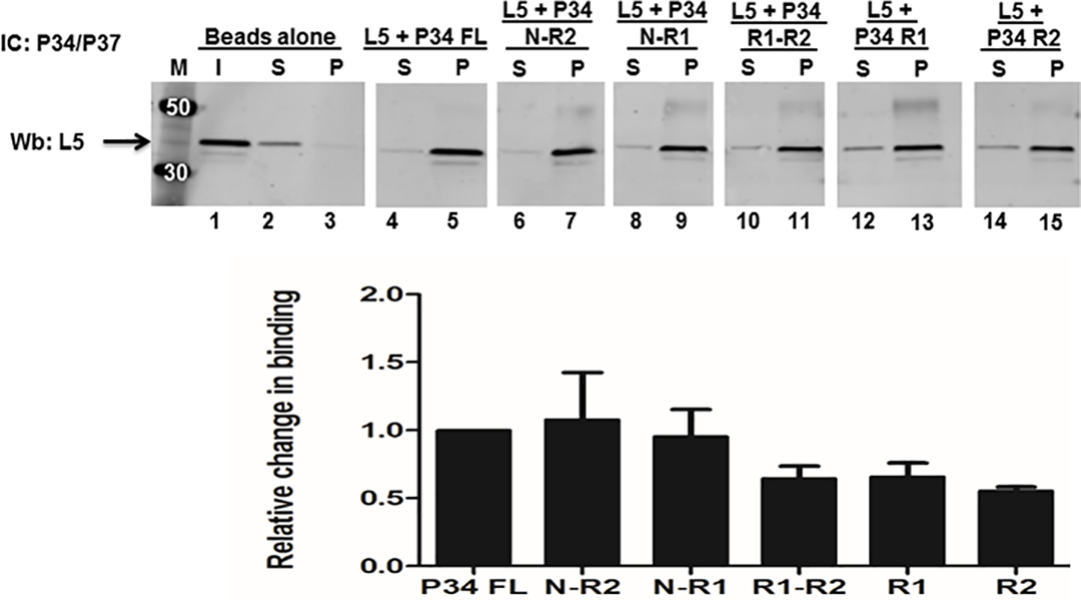
The N-terminus and RRM1 domains of P34 mediate the majority of the interaction between P34 and L5 protein. P34 or P34 truncated proteins were incubated with L5 and added to beads cross-linked with anti-P34/P37 antibody (IC: P34/P37) to capture P34 with L5 bound. The immune captured complexes were analyzed by western blotting (Wb) using anti-L5 antibody (L5 = 35 kDa). M = molecular weight marker, Beads alone = no anti-P34/P37 antibody, I = Input (50% of total protein used in experiment, anti-L5 antibody control), S = supernatant (unbound L5), P = (bound L5). The blots were cropped to remove the input (I) lanes because they are redundant. The bar graph represents the mean of at least three biological replicates and the error bars represent the standard error of the mean (SEM).

Our laboratory previously showed that the N-terminus and RRM domains of P34 are involved in not only mediating the interaction between P34 and 5S rRNA but also with L5 protein [20]. Therefore, we used the same truncated P34 proteins from the studies with 5S rRNA (Fig 1) in immune capture assays in order to characterize how these domains mediate protein-protein interactions with L5. The immune captured complexes were analyzed by western blotting using anti-L5 antibody (Fig 3. The bar graphs represent the mean and standard error of the mean (SEM) on at least three biological replicates).

Result from these experiments showed that similar amount of L5 was bound to the P34 protein lacking the C-terminus (1.1) (Fig 3 lane 7) relative to the amount of L5 that bound to the P34 full-length protein (set as 1) (Fig 3 lane 5). Also a similar amount of L5 was bound to the P34 truncate protein lacking both RRM2 and the C-terminus (0.93) (Fig 3 lane 9) relative to the amount of L5 that bound to the P34 full-length protein (Fig 3 lane 5). Therefore, from these two experiments we concluded that both RRM2 and the C-terminus of P34 do not contribute to the binding between P34 and L5. There was approximately a two-fold decrease in the amount of L5 bound to the RRM domains alone (0.64), (Fig 3 lane 11) and also in the amount of L5 bound to the P34 RRM1 truncate protein alone (0.65) (Fig 3 lane 13) relative to the amount of L5 bound to the P34 full-length protein (Fig 3 lane 5). Collectively, these results suggest that the N-terminus of P34 contributes more to the binding with L5 than either RRM2 or the C-terminus (Fig 3. compare lanes 7, 9, and 11). There was also a two-fold decrease in the amount of L5 that was bound to the P34 RRM2 truncate protein alone (0.54) (Fig 3 lane 15) relative to the amount of L5 bound to the P34 full-length protein (Fig 3 lane 5). This shows that the RRM2 construct alone is able to bind L5. However, in the presence of the N-terminus and RRM1 domains of P34, its contribution to binding with L5 is negligible. Since the results of the immune capture experiments between L5 and P34 truncates containing the N-terminus and RRM1 were similar, we concluded that these are the domains that mediate the most binding with L5 (Fig 3. compare lanes 7, 9,11 and 13).

### Mutation of the aromatic amino acids of the RNP1 and RNP2 consensus sequences in P34 lead to a modest increase in the binding affinity of 5S rRNA for P34

**Fig 4:**
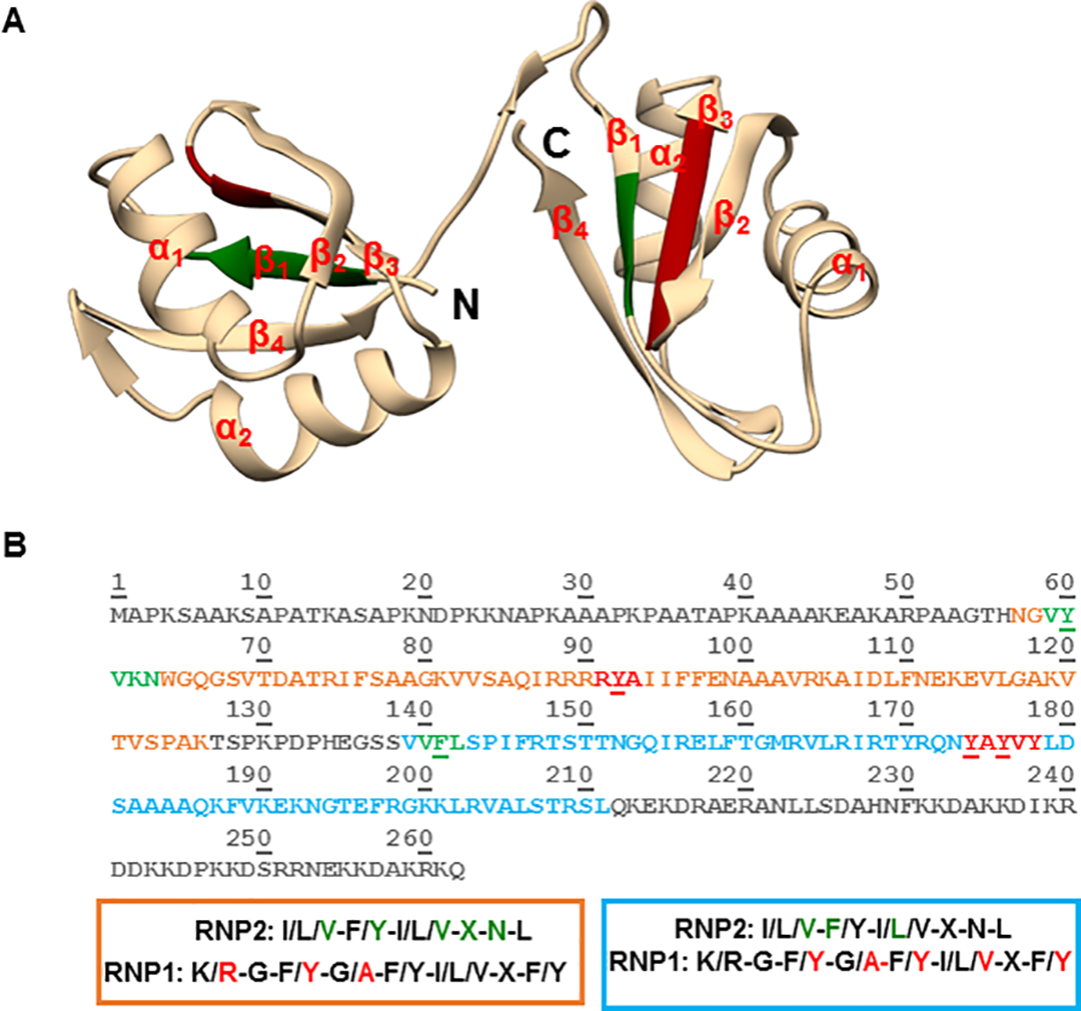
Analysis of the primary sequence and predicted tertiary structure of P34 protein. **Panel A.** Predicted tertiary structure of the RNA recognition motifs (RRMs) domains of P34. P34 is predicted to contain two RRM domains connected by a short linker. The RRMs are composed of four anti-parallel β-stands packed against two α-helices that fold into a βαββαβ topology. N = N-terminus, C = C-terminus, green = location of the RNP2 residues in the predicted structure, red = location of RNP1 residues in the predicted structure. **Panel B.** Primary sequence of P34. orange residues = RRM1, blue residues = RRM2, green residues = RNP2, red residues = RNP1, underlined residues = conserved aromatic amino acids. The colored boxes (orange = RRM1, blue = RRM2) depict the full sequences that compose the consensus RNP1 and RNP2 sequences with the colored green (RNP2) and red (RNP1) residues showing the conserved residues in both of the RRM domains of P34 protein.

In addition to possessing a canonical tertiary structure, RRM domains are also defined by the presence of two consensus RNP1 and RNP2 sequences in the β1 and β3 strands that mediate binding with their RNA target [21, 22]. RNP2 is composed of six amino acid residues ([I/L/V]-[F/Y]-[I/L/V]-X-N-L) and is located approximately 30 residues N-terminal to RNP1 which is composed of eight amino acid residues ([K/R]-G-[F/Y]-[G/A]-[F/Y]-[I/L/V]-X-[F/Y]) [24]. The aromatic residues in position 2 of RNP2 and in positions 3 and 5 of RNP1 have been specifically described to mediate base stacking interactions with the RNA target and in maintaining the hydrophobic surface of the β-sheet [23, 25, 26]. We used the tertiary structure prediction program I-TASSER [27–29] and chimera [30] to show that the two RRM domains of P34 are predicted to be canonical RRM domains composed of four anti-parallel β-strands packed against two α-helices that fold in the βαββαβ topology (Fig 4. Panel A).

**Fig 5:**
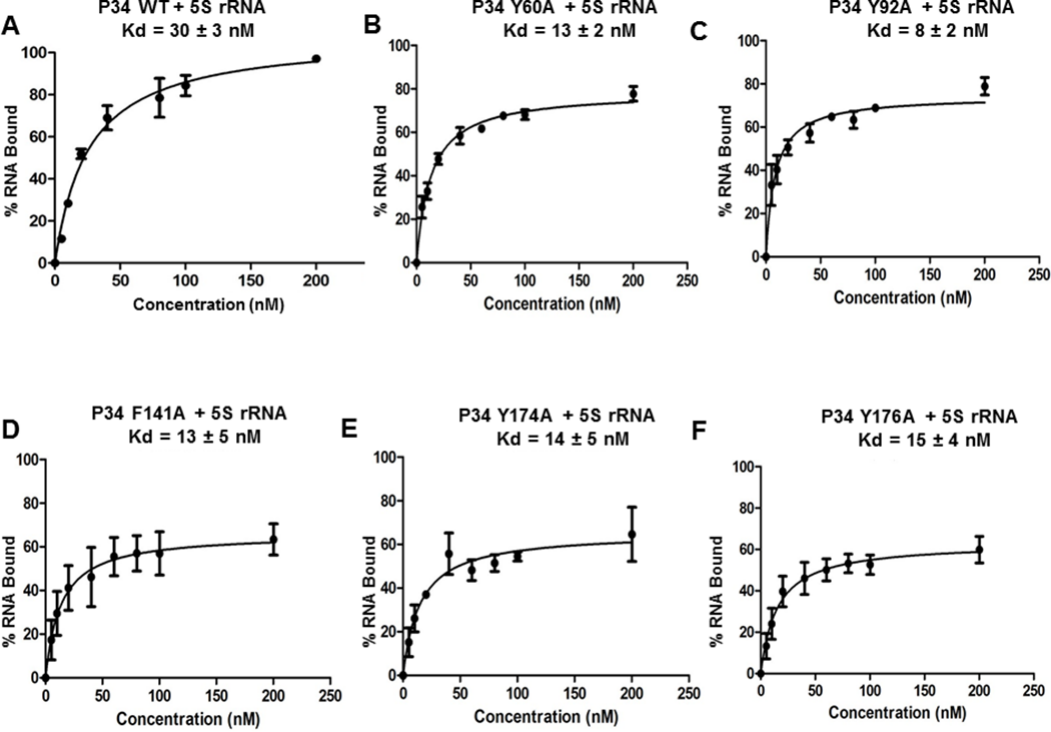
Mutation of the aromatic amino acids in RNP1 and RNP2 of P34 leads to an increase in the binding affinity between 5S rRNA and P34. Recombinant proteins were incubated with radiolabeled 5S rRNA and their interactions were analyzed using filter binding assays. The values obtained from the quantification of the bound and free 5S rRNA (bound/[bound + free]) were used to calculate the binding affinities (Kd) of 5S rRNA for the P34 wild type (WT) protein (positive control) and for the mutated P34 proteins.

Analysis of the primary sequence of the RRM domains of P34 showed that each of the RRMs contains partially conserved RNP1 and RNP2 consensus sequences (Fig 4. Panel B, RNP2 residues in green, RNP1 residues in red). We observed that the degree of conservation of the RNP sequences is different for the two RRM domains. Furthermore, we observed that for each of the RNP1 and RNP2 sequences in both of the P34 RRM domains, the aromatic amino acids are conserved in their respective positions (Fig 4. Panel B, aromatic amino acids are underlined). Studies have shown that mutation of these residues and/or mutations of the other residues in RNP1 and RNP2 have led to a decrease in binding between the mutated protein and its RNA binding target [26, 31]. Therefore, in order to determine whether the aromatic residues in RNP1 and RNP2 of the P34 RRM domains are involved in mediating binding between P34 and 5S rRNA, we mutated these residues to alanine because it is an amino acid that is smaller compared to the aromatic residues, neutral in terms of mediating binding, and is hydrophobic. If the aromatic residues are involved in direct binding to 5S rRNA, mutating them to alanine would be able to disrupt the interactions thereby decreasing the binding affinity of 5S rRNA for the mutated P34 protein. Surprisingly, the results from these experiments showed that the calculated binding affinity of 5S rRNA for all of the mutated P34 proteins increased by approximately two fold (Fig 5. Panels B-F) compared to the binding affinity of 5S rRNA for wild type (WT, non-mutated) P34 protein (Fig 5. Panel A). Therefore, mutating the conserved aromatic residues in RNP1 and RNP2 of P34 to alanine leads to a stronger interaction with 5S rRNA. This suggests that in P34 the conserved aromatic residues perform a non-canonical role in mediating interaction between 5S rRNA and P34. To further characterize these results, we used these mutated P34 proteins in immune capture experiments with L5 protein. The results from these experiments showed that some of the mutations bound to L5 in a similar manner to wild type non-mutated P34 protein (Y92A, Y174A, Y176A) while other mutations slightly decreased binding to L5 protein (Y60A, F141A). Thus, the observed effect of these mutated proteins on 5S rRNA binding is very specific, suggesting that they modulate binding to P34.

### Mutation of four arginine residues in RRM1 of P34 leads to a decrease in binding between P34 and L5

**Fig 6:**
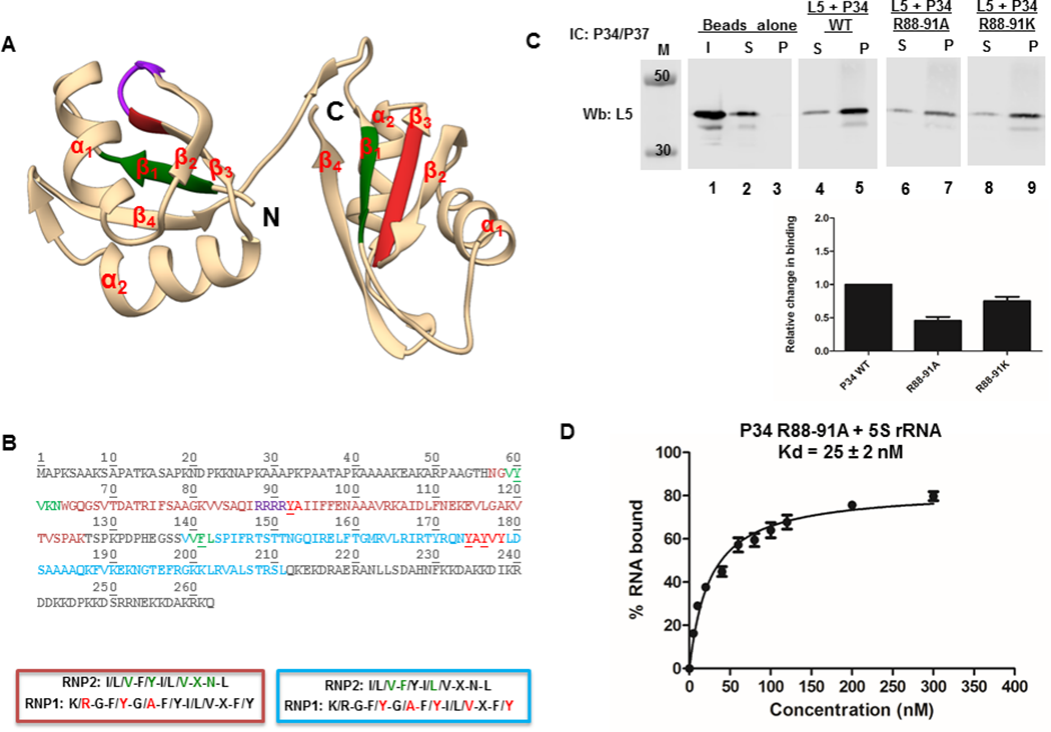
Mutation of four arginine residues in P34 leads to a decrease in binding between P34 and L5. **Panel A.** Predicted tertiary structure of the RRM domains of P34. P34 is predicted to contain two RRM domains connected by a short linker. The RRMs are composed of four anti-parallel β-stands packed against two α-helices that fold into a βαββαβ topology. N = N-terminus, C = C-terminus, green = location of the RNP2 residues in the predicted structure, red = location of RNP1 residues in the predicted structure, purple = R88-91 residues. **Panel B.** Primary sequence of P34. Orange residues = RRM1, blue residues = RRM2, green residues = RNP2, red residues = RNP1, Purple residues = location of the four arginine residues (R88-91) that were mutated to alanine, underlined residues = conserved aromatic amino acids. The colored boxes (orange = RRM1, blue = RRM2) depict the full sequences that compose the consensus RNP1 and RNP2 sequences with the colored green (RNP2) and red (RNP1) residues showing the conserved residues in both of the RRM domains of P34 protein. **Panel C**. P34 or mutated P34 proteins were incubated with L5 and added to beads cross-linked with anti-P34/P37 antibody (IC: P34/P37) to capture P34 with L5 bound. The immune captured complexes were analyzed by western blotting (Wb) using anti-L5 antibody (L5 = 35 kDa). M = molecular weight marker, Beads alone = no anti-P34/P37 antibody, I = Input (100% of total protein used in experiment, anti-L5 antibody control), S = supernatant (unbound L5), P = (bound L5). The blots were cropped to remove the input (I) lanes because they are redundant. The bar graph represents the mean of at least three biological triplicates and the error bars represent the standard error of the mean (SEM). **Panel D.** Recombinant P34 and P34 R88-91A proteins were incubated with radiolabeled 5S rRNA and their interactions were analyzed using filter binding assays. The values obtained from the quantification of the bound and the free 5S rRNA (bound/[bound +free]) were used to calculate the binding affinity (Kd) of 5S rRNA for the P34 non-mutated protein and for the P34 R88-91A mutated protein using Graphpad prism.

The RRM domains of P34 are involved in mediating both protein-RNA and protein-protein interactions. In order to identify specific residues that mediate direct interactions with L5 protein, we hypothesized that since the β-sheet of the RRM domains are occupied with RNA, the α-helices of the RRM domains might contain residues that mediate binding with L5. From analysis of the primary sequence of the predicted α-helices of P34, we did not observe any signature motifs characterized to mediate protein-protein interactions. To identify which residues are important for binding with L5, we mutated charged residues (arginine, lysine, aspartate, and glutamate) in the α -helices of the P34 RRM domains to alanine in order to disrupt the electrostatic interactions between P34 and L5. These residues were mutated to alanine due to the fact that it is a small non-charged residue, which would disrupt binding (if mediated by charge) between P34 and L5. However, mutation of these residues did not have an effect on the interaction between P34 and L5. We then mutated four arginine residues (P34 R88-91A, R91A is part of RNP1 in RRM1) that are not located in the predicted α-helices but are located between the predicted β_2_-β_3_ strands of RRM1 to alanine (Fig 6. Panel A purple region, Panel B purple residues). Results from the immune capture experiment showed that there was a two-fold decrease in the amount of L5 that was bound to the P34 R88-91A mutated protein (0.45) relative to the control reaction (L5 + P34 WT, set ass 1) (Fig 6. Panel C compare lane 5 and lane 7. The bar graphs represent the mean and standard error of the mean (SEM) on at least three biological replicates).

This result can either be interpreted as showing that these residues are those that are critical in mediating the interaction between P34 and L5 or that the mutation of these four arginine residues to alanine caused a change in the structure of the P34 protein. To dissect this result further, filter binding assays were performed between the mutated P34 R88-91A protein and 5S rRNA. This mutation did not cause a change in the binding affinity of 5S rRNA for P34 (Fig 6. Panel D). This suggests that the decrease in binding to L5 was not due to a misfolded or a non-functional P34 protein. These four arginine residues were then mutated to lysine (P34 R88-91K) and the effect that these mutations had on the interaction between P34 and L5 were analyzed using immune capture assay. Compared to the control reaction, there was still a decrease in the amount of L5 that was bound to the P34 R88-91K mutated protein (0.75) (Fig 6. Panel C, lane 9. The bar graph represents the mean and standard error of the mean (SEM) on at least three biological replicates). Since the mutation to lysine, an amino acid similar to arginine in terms of size and charge, was unable to fully restore the binding between P34 and L5 to wild type levels, we concluded that the four arginine residues are critical in mediating interactions between P34 and L5.

### Mutation of RNP1 sequences in RRM1 of P34 leads to an increase in binding between P34 and 5S rRNA and between P34 and L5 protein

**Fig 7:**
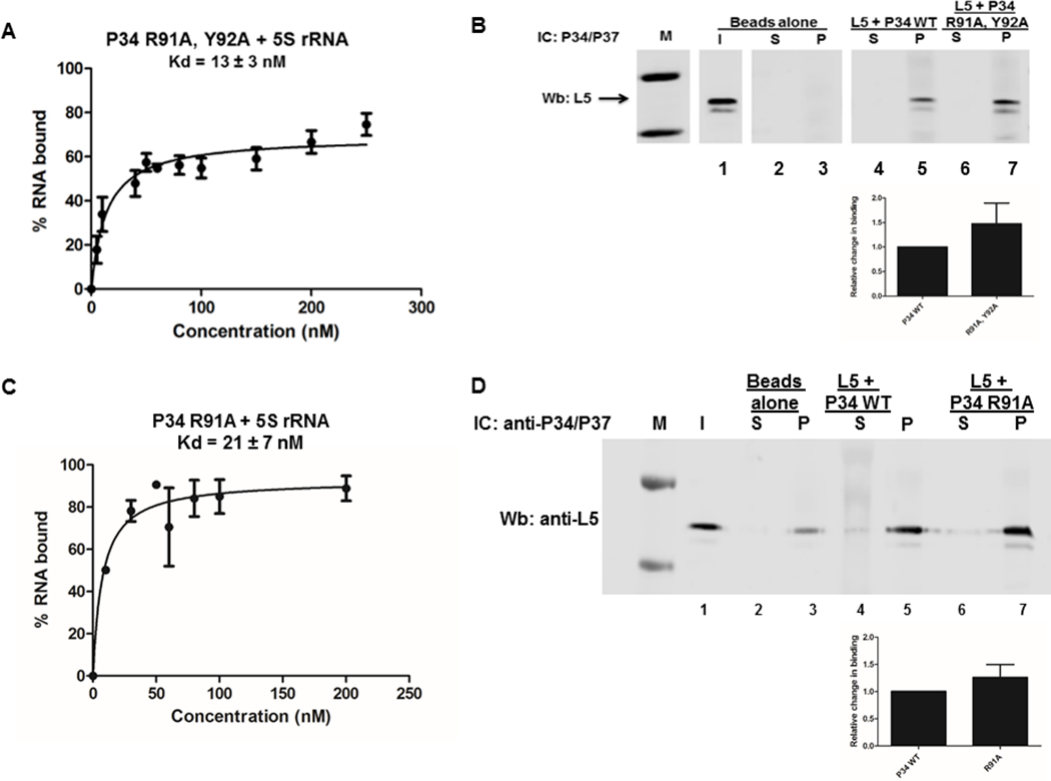
Mutation of the RNP1 residues in RRM1 of P34 leads to an increase in the binding between P34 and 5S rRNA and between P34 and L5. **Panel A**. Recombinant P34 R91A, Y92A mutated protein was incubated with radiolabeled 5S rRNA and their interactions were analyzed using filter binding assays. The values obtained from the quantification of the bound and the free 5S rRNA (bound/[bound +free]) were used to calculate the binding affinity (Kd) of 5S rRNA for the mutated P34 protein. **Panel B.** P34 mutated P34 proteins were incubated with L5 and added to beads cross-linked with anti-P34/P37 antibody (IC: P34/P37) to capture P34 with L5 bound. The immune captured complexes were analyzed by western blotting (Wb) using anti-L5 antibody (L5 = 35 kDa). M = molecular weight marker, Beads alone = no anti-P34/P37 antibody, I = Input (50% of total protein used in experiment, anti-L5 antibody control), S = supernatant (unbound L5), P = (bound L5). The blots were cropped to remove the input (I) lanes because they are redundant. The bar graph represents the mean of the triplicate experiments and the error bars represent the standard error of the mean (SEM). **Panel C.** Recombinant P34 R91A, mutated protein was incubated with radiolabeled 5S rRNA and their interactions were analyzed using filter binding assays. **Panel D.** P34 R91A mutated protein was incubated with L5 and added to beads cross-linked with anti-P34/P37 antibody (IC: P34/P37) to capture P34 with L5 bound. The immune captured complexes were analyzed by western blotting (Wb) using anti-L5 antibody (L5 = 35 kDa). The bar graph represents the mean of the triplicate experiments and the error bars represent the standard error of the mean (SEM).

In these studies we showed that the N-terminus and RRM1 domains of P34 are strongly involved in mediating the binding to both 5S rRNA and L5 and that mutation of aromatic amino acids in RNP1 and 2 in RRM1 leads to an increase in the binding of 5S rRNA to P34. We also showed that one of the residues that affect binding to L5 is part of RNP1 (Figs 4 and 6. Panels A and B, R91 part of R88-91A). These results suggest that the binding site for both 5S rRNA and L5 are close to each other. Therefore, we hypothesized that the RNP1 residues in the RRM1 domain of P34 regulate both 5S rRNA and L5 binding to P34. To test this hypothesis we mutated the residues of RNP1 in RRM1 to alanine and then characterized how this mutated P34 protein interacts with 5S rRNA and L5. Results from the filter binding assay between P34 R91A, Y92A and 5S rRNA showed that the binding affinity of 5S rRNA for this mutated protein increased two fold compared to the binding affinity of 5S rRNA for the wild type (non-mutated) P34 protein (Fig 7. Panel A). This is similar to the result observed when just the aromatic amino acid (Y92) in RNP1 was mutated to alanine (Fig 5. Panel C). The immune capture experiment between L5 and the P34 R91A, Y92A mutated protein showed that slightly more of L5 was bound to this mutated protein (1.5) compared to the amount that bound to the wild type non-mutated P34 protein (set as 1) (Fig 7 Panel B, compare lane 5 and lane 7. The bar graphs represent the mean and standard error of the mean (SEM) on three biological replicates). This was unexpected due to the fact that the R91 residue is part of the four tandem arginine residues that when mutated to alanine significantly decreased the binding between P34 and L5 (Fig 6. Panel C, lanes 7 and 9). We also tested the effect that the P34 R91 residue alone has on the interaction with 5S rRNA and L5 protein. We mutated this residue to alanine and used it in filter binding and immune capture assays. Results from the filter binding experiments showed that, compared to the binding affinity of 5S rRNA for wild type (non-mutated) P34 protein, mutation of R91 to alanine had no significant effect on the binding affinity of 5S rRNA for this mutated P34 protein (Fig 7. Panel C). Results from the immune capture assays showed that slightly more L5 bound to the P34 R91A mutated protein (1.3) relative to the amount that bound to non-mutated P34 protein (Fig 7. Panel D, compare lanes 5 and 7. The bar graphs represent the mean and standard error of the mean (SEM) on three biological replicates). These results show that the Y92A residue is the one that is influencing 5S rRNA binding to P34. Both R91 and Y92 influence L5 binding; however, their effects on binding are not additive when both residues are mutated to alanine. These results support our hypothesis that the role of the RNP1 residues in RRM1 is to modulate both 5S rRNA and L5 binding.

## Discussion

Much of the literature concerning RRM domains focuses on RRM domain containing proteins that are involved in eukaryotic splicing and mRNA metabolism [32]. In *T*. *brucei*, many RNA binding proteins containing RRM domains have been identified and characterized [33–35]. However, the mechanism by which these RRM domains mediate binding has not been described. Our laboratory has characterized the interactions mediated by two trypanosome-specific RNA binding proteins P34 and P37 and their involvement in ribosome biogenesis. Both of these proteins contain two RRM domains that are predicted to fold in the canonical βαββαβ topology. Previous P34 protein studies have shown that the RRM domains are involved in binding to both 5S rRNA and L5 protein [19, 20]. RRM domains are the most abundant and most studied RNA binding domain [32]. Although the canonical structure of the RRM domain is conserved, this domain has developed diverse mechanisms to not only mediate protein-RNA interactions but also protein-protein interactions [32, 36]. In some instances, in order to mediate both protein-RNA and protein-protein interactions, the RNP1 and RNP2 consensus sequences are absent or only partially conserved and there is usually a signature protein binding region in addition to the RRM domain. This has been characterized for the U2AF homology motif (UHM) proteins, such as U2AF^65^, which contains a UHM domain (R-X-F) that mediates protein-protein interactions [37]. This has also been shown for two proteins involved in splicing, SRp20 and 9G8, which contain serine-arginine repeats located C-terminal to the RRM domain that meditates protein-protein interactions [38]. Several studies have also identified RRM domains can mediate simultaneous binding to both protein and RNA [36]. These examples show that there is a range of mechanisms for recognition by RRM domains. Therefore, the only way to fully characterize how an RRM domain mediates specific binding to its target molecule is by studying its interactions. Analysis of the primary sequence of P34 protein shows that it contains the canonical RNA binding motif in its RRM domains; however, we did not observe any protein binding motifs similar to those described above. Therefore, in these studies we explored how the RRM domains of P34 might mediate both interactions with 5S rRNA and L5 protein.

Using fluorescence resonance energy transfer (FRET), our laboratory previously characterized how the P34 N-terminal domain alone, the P34 RRM domains alone, and the P34 C-terminal domain alone mediate binding with 5S rRNA and with L5 [19, 20]. In the described studies here, we have dissected those results further by specifying how each of the domains of P34 contribute to binding to 5S rRNA and L5. For the protein-RNA interactions, we determined that the RRM1 domain of P34 mediates most of the binding between P34 and 5S rRNA since all of the P34 truncates containing RRM1 had similar calculated binding affinities (Fig 2). We determined that the C-terminus of P34 contributes to binding with 5S rRNA because in its absence, the binding affinity of 5S rRNA for P34 decreased two fold. However, from previously published results, the C-terminus alone is unable to bind to 5S rRNA [19]. We also showed that the N-terminus, when combined with RRM1 alone has a small negative effect on 5S rRNA binding. However, this negative effect is not observed in the presence of RRM2 (Fig 2. Panel B and 2 Panel C) even though RRM2 alone binds poorly to 5S rRNA (Fig 2. Panel F). These results indicate that these regions do not bind but modulate binding of 5S rRNA to P34. Unlike the protein-RNA results, for the protein-protein interactions between P34 and L5 we determined that the N-terminus of P34 combined with RRM1 alone mediates most of the binding with L5 (Fig 3. lane 7 and lane 9) We also showed that the C-terminus of P34 does not contribute to binding with L5 and although the P34 RRM2 domain alone is able to bind to L5, it does not appear to contribute to binding in the presence of the N-terminus and RRM1 (Fig 3. compare lanes 7, 11, and 15).

Collectively, our results suggest that the N-terminus and RRM1 domains of P34 mediate residue specific binding to 5S rRNA and L5 and that the C-terminus and RRM2 structurally enhance these binding interactions. Several studies have described the involvement of the N- and/or C-terminus of RRM domain containing proteins in structurally contributing to the binding with the RNA target [39, 40]. NMR studies of the unfolding and folding patterns of the cleavage stimulation factor-64 (Cstf-64) protein showed that when this protein is not bound to its RNA target, the C-terminus adopts a helical structure and covers the β-sheet, which prevents random RNA binding. In the presence of its RNA target (GU nucleotides) the C-terminal helix unfolds and the β-sheet undergoes conformational changes upon specific RNA binding [41]. In our studies the N- and C- termini may function in a similar way. There may be a conformation of P34 that is preferable for both 5S rRNA and L5 binding and this conformation is dependent on the specific binding partner. For the interaction between P34 and 5S rRNA the N-terminus may fold in a conformation that limits 5S rRNA binding but does not affect L5 binding. Also, both the C-terminus and RRM2 domain of P34 may fold in a conformation that is favorable for the simultaneous binding of 5S rRNA and L5 to P34. Proteins that contain multiple RRM domains have been shown either to use more than one of the RRM domains to mediate RNA binding, as shown for the TIA-1 protein [42] or to utilize each of the RRM domains for a specific purpose, as shown for the UA1 protein [26, 43–45]. In the case of the P34 domains, our results suggest that all of the domains are important for binding in that the N-terminus and RRM1 contribute the most to the interaction with 5S rRNA and L5 and RRM2 and the C-terminus function to modulate these interactions.

Studies of canonical RRM-RNA interactions have shown that the RNP1 and RNP2 consensus sequences are involved in mediating these interactions [32]. Although not all of the sequences that compose RNP1 and RNP2 are conserved in the RRMs of P34, the aromatic amino acids described to mediate RNA binding are conserved. Therefore, we mutated these aromatic residues to determine whether the mutations would disrupt P34 and 5S rRNA binding. These mutations led to a two-fold increase in the binding affinity of 5S rRNA for P34 instead of decreasing the affinity (Fig 5). This was a surprising result because in several studies where the residues in RNP1 and RNP2 have been mutated, these mutations lead to a strong decrease in the binding affinity of the RNA target for the protein [25, 31, 46, 47]. To our knowledge, this is the first report indicating that mutation of these conserved aromatic residues to alanine increases the binding affinity of the target RNA for the protein. Although unexpected, these results show that similar to other RRM-RNA binding mechanisms that have been described in literature, the residues that confer specificity of 5S rRNA binding to P34 may be located in regions outside of the RNP1 and RNP2 consensus sequences. These aromatic residues could be important in maintaining structure instead of making residue specific contact with 5S rRNA. This has been shown for the L5 protein where mutation of one aromatic residue (F180) to leucine led to a two-fold decrease in binding to 5S rRNA. However, mutation of the same residue to alanine led to a three-fold increase in the binding affinity of 5S rRNA for this mutated protein [48, 49].

The P34 RRM- mediated binding interaction with L5 protein is trypanosome-specific. Thus, since P34 does not contain any signature protein-binding motif, to identify specific residues in P34 that mediate binding with L5, we individually mutated charged residues in the α-helices of P34. Our rationale was that the helices in the RRM domains would not be occupied by 5S rRNA and therefore could contain residues that mediate L5 binding. Mutation of these residues had no effect on binding between P34 and L5. In fact, the only residues that when mutated to alanine significantly decreased the interaction between P34 and L5 were four tandem arginine residues (R88-91) that are located in a predicted region between β_2_—β_3_ (the last arginine, R91, is part of RNP1) (Fig 6 Panel A and B). However, mutation of these residues had no effect on the binding affinity of 5S rRNA for P34 protein. Thus, unlike 5S rRNA binding to P34, L5 binding to P34 is sequence specific and these sequences lie in the junction of the predicted RNP1-linker region.

In eukaryotes, L5 is the only ribosomal protein that binds to 5S rRNA outside of the pre-ribosome. This interaction functions to stabilize and localize 5S rRNA to the nucleolus to join ribosome assembly [50]. In the absence of L5, 5S rRNA is unable to localize to the nucleolus and does not get assembled into the forming pre-ribosomes, which leads to defects in proper ribosome formation [5]. Studies have also shown that the proper formation of this complex functions as one of the regulators of ribosome biogenesis [6, 7]. Our laboratory has shown that P34 and P37 are also essential for stability of 5S rRNA and that L5 alone is not sufficient to maintain wild type levels of 5S rRNA in the absence of P34 and P37 [14]. Hence P34 and P37 are crucial for complex formation in *T*. *brucei*. In characterizing how P34 mediates binding to both 5S rRNA and L5, conclusions drawn from both mutational analyses of P34 suggest that the binding site on P34 for both 5S rRNA and L5 are near each other in the N-terminus and RRM1 domains of P34. Since the formation of this complex is crucial for proper ribosome assembly, we hypothesized that the close proximity of the binding sites of both L5 and 5S rRNA on P34 is a mechanism for P34 to regulate/ mediate simultaneous binding to these two factors. More specifically, we hypothesized that the RNP1 residues in the RRM1 domain of P34 are part of the residues that are involved in modulating the binding of 5S rRNA and L5 to P34 protein. We showed that mutation of these RNP1 residues together led to a two-fold increase in the binding between this mutated protein with 5S rRNA (Fig 7 panel A) but the P34 R91 residue alone had no effect on 5S rRNA binding when mutated to alanine (Fig 7. Panel C). Mutation of the RNP1 residues together and individually had a small increase in binding with L5 protein (Fig 7 panels B and D). These results are similar to the result observed when we mutated only the tyrosine (Y92) to alanine (Fig 5 Panel C). These results show that the contribution of RNP1 residues to binding is not additive. Therefore, it could be that these RNP1 residues are involved in maintaining a specific structure that is favorable for simultaneous binding of L5 and 5S rRNA.

As previously mentioned, several studies have described RRM mediated simultaneous protein-protein and protein-RNA interactions. One example is from NMR studies of the polypyrimidine tract binding protein (PTB) a regulatory splicing repressor, which showed that the RRM2 domain of PTB mediates simultaneous binding with Raver1 protein and its RNA target sequence [51]. Studies of the interaction between the p14 protein, a member of the splicesomal U2 and U11/U12 small nuclear ribonucleoprotein (snRNP), with another U2 snRNP protein, SF3b155, showed that this protein- protein interaction limited RNA binding [52]. p14 contains only one RRM domain where the majority of the β-sheet is covered by the SF3b155 protein leaving only several binding sites (one containing a conserved RNP2 aromatic amino acid) to mediate weak interactions with its RNA target [52]. Our studies of the interactions of the RRM domains of P34 with 5S rRNA and L5 suggest that RRM-RNA interaction is structure- specific and that RRM-protein interaction is sequence-specific. These results led us to hypothesize that modulation of binding to P34 is necessary for proper complex formation. We offer the model that this modulation is mediated by the RNP1 sequences in RRM1 and residues flanking this sequence to ensure that there is one conformation of P34 that is favorable for simultaneous binding of both 5S rRNA and L5. Whereas L5 binding of P34 is mediated largely by the four arginine residues adjacent to RNP1 in RRM1, specific 5S rRNA binding could be mediated by the final structure that P34 forms upon simultaneous binding with both 5S rRNA and L5. To test the hypotheses formulated from these studies, future work in our laboratory will focus on characterizing these simultaneous interactions between all three of the factors (5S rRNA-L5-P34). These experiments, together with structural studies of P34 will allow for us to be able to test our model on the role of the RNP1 and RNP2 residues in P34 and also on the role of the RRM2 and C-terminal domains of P34. Collectively these experiments will allow for us to determine whether the binding of P34 with 5S rRNA and L5 are due to conformational selection, induced fit binding, or both.

## Supporting information

S1 Fig Representative Coomassie stained gels and western blot images of the P34 proteins used in this study. For the Commassie stain 10 μg of protein was analyzed and 1 μg of protein was analyzed for the western blot using anti–P34/P37 antibody. **Panel A**. Coomassie and western blot analysis of the P34 truncated proteins. Lane 1 = P34, lane 2 = N-R2, lane 3 = N-R1, lane 4 = R1 lane 5 = R1-R2, lane 6 = R2 (the difference in migration pattern is due to different gels (4–12% gradient or 10%) being used for protein analysis). The molecular weight for the P34 truncated proteins are as follows: P34 = 34 kDa, P34 N-R2 = 23 kDa, N-R1 = 14 kDa, R1 = 8.7 kDa. R1-R2 = 17 kDa, R2 = 8.8 kDa. **Panel B.** Coomassie and western blot analysis of P34 and P34 RNP mutated proteins. Lane 1 = P34, lane 2 = P34 Y60A, lane 3 = P34 Y92A, lane 4 = P34 F141A, lane 5 = P34 Y174A, lane 6 = P34 Y176A. The molecular weight for both P34 and the mutated P34 proteins is 34 kDa. **Panel C:** Coomassie and western blot analysis of P34 and P34 mutated proteins. Lane 1 = P34 protein, lane 2 = P34 R91A, lane 3 = P34 R91A, Y92A, lane 4 = P34 R88-91K, lane 5 = P34 R88-91A. The molecular weight for both P34 and the mutated P34 proteins is 34 kDa.
(TIF)

S2 Fig Representative membranes of the filter binding experiments performed in this study. Nitrocellulose membranes were used to capture the protein-RNA complex and the nytran membranes were used to capture free unbound RNA.
(TIF)

## References

[1] Wilson DN, Doudna Cate JH. The structure and function of the eukaryotic ribosome. Cold Spring Harb Perspect Biol. 2012; 4:10.1101/cshperspect.a011536 PMC3331703 22550233

[2] Gamalinda M, Ohmayer U, Jakovljevic J, Kumcuoglu B, Woolford J, Mbom B, et al A hierarchical model for assembly of eukaryotic 60S ribosomal subunit domains. Genes Dev. 2014; 28: 198–210 doi: 10.1101/gad.228825.113 24449272 10.1101/gad.228825.113 PMC3909792

[3] Umaer MC K., Williams N.. (2016) Ribosomal RNA Processing. In Encyclopedia of Cell Biology 288–96.

[4] Fatica A, Tollervey D. Making ribosomes. Curr Opin Cell Biol. 2002; 14: 313–8 12067653 10.1016/s0955-0674(02)00336-8

[5] Ciganda M, Williams N. Eukaryotic 5S rRNA biogenesis. Wiley Interdiscip Rev RNA. 2011; 2: 523–33 doi: 10.1002/wrna.74 21957041 10.1002/wrna.74 PMC3278907

[6] Zhang J, Harnpicharnchai P, Jakovljevic J, Tang L, Guo Y, Oeffinger M, et al Assembly factors Rpf2 and Rrs1 recruit 5S rRNA and ribosomal proteins rpL5 and rpL11 into nascent ribosomes. Genes Dev. 2007; 21: 2580–92 doi: 10.1101/gad.1569307 17938242 10.1101/gad.1569307 PMC2000323

[7] Madru C, Lebaron S, Blaud M, Delbos L, Pipoli J, Pasmant E, et al Chaperoning 5S RNA assembly. Genes Dev. 2015; 29: 1432–46 doi: 10.1101/gad.260349.115 26159998 10.1101/gad.260349.115 PMC4511217

[8] Asano N, Kato K, Nakamura A, Komoda K, Tanaka I, Yao M. Structural and functional analysis of the Rpf2-Rrs1 complex in ribosome biogenesis. Nucleic Acids Res. 2015; 43: 4746–57 doi: 10.1093/nar/gkv305 25855814 10.1093/nar/gkv305 PMC4482071

[9] Kharde S, Calvino FR, Gumiero A, Wild K, Sinning I. The structure of Rpf2-Rrs1 explains its role in ribosome biogenesis. Nucleic Acids Res. 2015; 43: 7083–95 doi: 10.1093/nar/gkv640 26117542 10.1093/nar/gkv640 PMC4538828

[10] Lafontaine DL, Tollervey D. The function and synthesis of ribosomes. Nat Rev Mol Cell Biol. 2001; 2: 514–20 doi: 10.1038/35080045 11433365 10.1038/35080045

[11] Franco JR, Simarro PP, Diarra A, Jannin JG. Epidemiology of human African trypanosomiasis. Clin Epidemiol. 2014; 6: 257–75 doi: 10.2147/CLEP.S39728 25125985 10.2147/CLEP.S39728 PMC4130665

[12] Matthews KR. 25 years of African trypanosome research: From description to molecular dissection and new drug discovery. Mol Biochem Parasitol. 2015; 200: 30–40 doi: 10.1016/j.molbiopara.2015.01.006 25736427 10.1016/j.molbiopara.2015.01.006 PMC4509711

[13] Umaer K, Ciganda M, Williams N. Ribosome biogenesis in african trypanosomes requires conserved and trypanosome-specific factors. Eukaryot Cell. 2014; 13: 727–37 doi: 10.1128/EC.00307-13 24706018 10.1128/EC.00307-13 PMC4054266

[14] Hellman KM, Ciganda M, Brown SV, Li J, Ruyechan W, Williams N. Two trypanosome-specific proteins are essential factors for 5S rRNA abundance and ribosomal assembly in *Trypanosoma brucei*. Eukaryot Cell. 2007; 6: 1766–72 doi: 10.1128/EC.00119-07 17715362 10.1128/EC.00119-07 PMC2043393

[15] Zhang J, Williams N. Purification, cloning, and expression of two closely related *Trypanosoma brucei* nucleic acid binding proteins. Mol Biochem Parasitol. 1997; 87: 145–58 9247926 10.1016/s0166-6851(97)00060-1

[16] Ciganda M, Prohaska K, Hellman K, Williams N. A novel association between two trypanosome-specific factors and the conserved L5-5S rRNA complex. PLoS One. 2012; 7: e41398 doi: 10.1371/journal.pone.0041398 22859981 10.1371/journal.pone.0041398 PMC3409183

[17] Ciganda M, Williams N. Characterization of a novel association between two trypanosome-specific proteins and 5S rRNA. PLoS One. 2012; 7: e30029 doi: 10.1371/journal.pone.0030029 22253864 10.1371/journal.pone.0030029 PMC3257258

[18] Prohaska K, Williams N. Assembly of the *Trypanosoma brucei* 60S ribosomal subunit nuclear export complex requires trypanosome-specific proteins P34 and P37. Eukaryot Cell. 2009; 8: 77–87 doi: 10.1128/EC.00234-08 18723605 10.1128/EC.00234-08 PMC2620753

[19] Wang L, Ciganda M, Williams N. Defining the RNA-protein interactions in the trypanosome preribosomal complex. Eukaryot Cell. 2013; 12: 559–66 doi: 10.1128/EC.00004-13 23397568 10.1128/EC.00004-13 PMC3623441

[20] Wang L, Ciganda M, Williams N. Association of a novel preribosomal complex in *Trypanosoma brucei* determined by fluorescence resonance energy transfer. Eukaryot Cell. 2013; 12: 322–9 doi: 10.1128/EC.00316-12 23264640 10.1128/EC.00316-12 PMC3571310

[21] Muto Y, Yokoyama S. Structural insight into RNA recognition motifs: versatile molecular Lego building blocks for biological systems. Wiley Interdiscip Rev RNA. 2012; 3: 229–46 doi: 10.1002/wrna.1107 22278943 10.1002/wrna.1107

[22] Perez-Canadillas JM, Varani G. Recent advances in RNA-protein recognition. Curr Opin Struct Biol. 2001; 11: 53–8 11179892 10.1016/s0959-440x(00)00164-0

[23] Auweter SD, Oberstrass FC, Allain FH. Sequence-specific binding of single-stranded RNA: is there a code for recognition? Nucleic Acids Res. 2006; 34: 4943–59 doi: 10.1093/nar/gkl620 16982642 10.1093/nar/gkl620 PMC1635273

[24] Kishore S, Luber S, Zavolan M. Deciphering the role of RNA-binding proteins in the post-transcriptional control of gene expression. Brief Funct Genomics. 2010; 9: 391–404 doi: 10.1093/bfgp/elq028 21127008 10.1093/bfgp/elq028 PMC3080770

[25] Nagai K, Oubridge C, Ito N, Avis J, Evans P. The RNP domain: a sequence-specific RNA-binding domain involved in processing and transport of RNA. Trends Biochem Sci. 1995; 20: 235–40 7543225 10.1016/s0968-0004(00)89024-6

[26] Nagai K, Oubridge C, Jessen TH, Li J, Evans PR. Crystal structure of the RNA-binding domain of the U1 small nuclear ribonucleoprotein A. Nature. 1990; 348: 515–20 doi: 10.1038/348515a0 2147232 10.1038/348515a0

[27] Zhang Y. I-TASSER server for protein 3D structure prediction. BMC Bioinformatics. 2008; 9: 40 doi: 10.1186/1471-2105-9-40 18215316 10.1186/1471-2105-9-40 PMC2245901

[28] Yang J, Yan R, Roy A, Xu D, Poisson J, Zhang Y. The I-TASSER Suite: protein structure and function prediction. Nat Methods. 2015; 12: 7–8 doi: 10.1038/nmeth.3213 25549265 10.1038/nmeth.3213 PMC4428668

[29] Roy A, Kucukural A, Zhang Y. I-TASSER: a unified platform for automated protein structure and function prediction. Nat Protoc. 2010; 5: 725–38 doi: 10.1038/nprot.2010.5 20360767 10.1038/nprot.2010.5 PMC2849174

[30] Pettersen EF, Goddard TD, Huang CC, Couch GS, Greenblatt DM, Meng EC, et al UCSF Chimera—a visualization system for exploratory research and analysis. J Comput Chem. 2004; 25: 1605–12 doi: 10.1002/jcc.20084 15264254 10.1002/jcc.20084

[31] Lisbin MJ, Gordon M, Yannoni YM, White K. Function of RRM domains of *Drosophila melanogaster* ELAV: Rnp1 mutations and rrm domain replacements with ELAV family proteins and SXL. Genetics. 2000; 155: 1789–98 10924474 10.1093/genetics/155.4.1789 PMC1461190

[32] Daubner GM, Clery A, Allain FH. RRM-RNA recognition: NMR or crystallography…and new findings. Curr Opin Struct Biol. 2013; 23: 100–8 doi: 10.1016/j.sbi.2012.11.006 23253355 10.1016/j.sbi.2012.11.006

[33] Clayton C. The regulation of trypanosome gene expression by RNA-binding proteins. PLoS Pathog. 2013; 9: e1003680 doi: 10.1371/journal.ppat.1003680 24244152 10.1371/journal.ppat.1003680 PMC3820711

[34] Wurst M, Robles A, Po J, Luu VD, Brems S, Marentije M, et al An RNAi screen of the RRM-domain proteins of *Trypanosoma brucei*. Mol Biochem Parasitol. 2009; 163: 61–5 doi: 10.1016/j.molbiopara.2008.09.001 18840477 10.1016/j.molbiopara.2008.09.001

[35] Kolev NG, Ullu E, Tschudi C. The emerging role of RNA-binding proteins in the life cycle of *Trypanosoma brucei*. Cell Microbiol. 2014; 16: 482–9 doi: 10.1111/cmi.12268 24438230 10.1111/cmi.12268 PMC3974610

[36] Beckmann BM, Castello A, Medenbach J. The expanding universe of ribonucleoproteins: of novel RNA-binding proteins and unconventional interactions. Pflugers Arch. 2016; 468: 1029–40 doi: 10.1007/s00424-016-1819-4 27165283 10.1007/s00424-016-1819-4 PMC4893068

[37] Kielkopf CL, Lucke S, Green MR. U2AF homology motifs: protein recognition in the RRM world. Genes Dev. 2004; 18: 1513–26 doi: 10.1101/gad.1206204 15231733 10.1101/gad.1206204 PMC2043112

[38] Hargous Y, Hautbergue GM, Tintaru AM, Skrisovska L, Golovanov AP, Stevenin J, et al Molecular basis of RNA recognition and TAP binding by the SR proteins SRp20 and 9G8. EMBO J. 2006; 25: 5126–37 doi: 10.1038/sj.emboj.7601385 17036044 10.1038/sj.emboj.7601385 PMC1630407

[39] Nagai K, Oubridge C, Ito N, Jessen TH, Avis J, Evans P. Crystal structure of the U1A spliceosomal protein complexed with its cognate RNA hairpin. Nucleic Acids Symp Ser. 1995; 1–2 8841523

[40] Tsuda K, Someya T, Kuwasako K, Takahashi M, He F, Unzai S, et al Structural basis for the dual RNA-recognition modes of human Tra2-beta RRM. Nucleic Acids Res. 2011; 39: 1538–53 doi: 10.1093/nar/gkq854 20926394 10.1093/nar/gkq854 PMC3045587

[41] Deka P, Rajan PK, Perez-Canadillas JM, Varani G. Protein and RNA dynamics play key roles in determining the specific recognition of GU-rich polyadenylation regulatory elements by human Cstf-64 protein. J Mol Biol. 2005; 347: 719–33 doi: 10.1016/j.jmb.2005.01.046 15769465 10.1016/j.jmb.2005.01.046

[42] Wang I, Hennig J, Jagtap PK, Sonntag M, Valcarcel J, Sattler M. Structure, dynamics and RNA binding of the multi-domain splicing factor TIA-1. Nucleic Acids Res. 2014; 42: 5949–66 doi: 10.1093/nar/gku193 24682828 10.1093/nar/gku193 PMC4027183

[43] Mackereth CD, Sattler M. Dynamics in multi-domain protein recognition of RNA. Curr Opin Struct Biol. 2012; 22: 287–96 doi: 10.1016/j.sbi.2012.03.013 22516180 10.1016/j.sbi.2012.03.013

[44] Shamoo Y, Abdul-Manan N, Williams KR. Multiple RNA binding domains (RBDs) just don't add up. Nucleic Acids Res. 1995; 23: 725–8 7535921 10.1093/nar/23.5.725 PMC306750

[45] Dreyfuss G, Matunis MJ, Pinol-Roma S, Burd CG. hnRNP proteins and the biogenesis of mRNA. Annu Rev Biochem. 1993; 62: 289–321 doi: 10.1146/annurev.bi.62.070193.001445 8352591 10.1146/annurev.bi.62.070193.001445

[46] Jessen TH, Oubridge C, Teo CH, Pritchard C, Nagai K. Identification of molecular contacts between the U1 A small nuclear ribonucleoprotein and U1 RNA. EMBO J. 1991; 10: 3447–56 1833186 10.1002/j.1460-2075.1991.tb04909.x PMC453073

[47] McLaughlin KJ, Jenkins JL, Kielkopf CL. Large favorable enthalpy changes drive specific RNA recognition by RNA recognition motif proteins. Biochemistry. 2011; 50: 1429–31 doi: 10.1021/bi102057m 21261285 10.1021/bi102057m PMC3050080

[48] DiNitto JP, Huber PW. A role for aromatic amino acids in the binding of *Xenopus* ribosomal protein L5 to 5S rRNA. Biochemistry. 2001; 40: 12645–53 11601989 10.1021/bi011439m

[49] DiNitto JP, Huber PW. Mutual induced fit binding of *Xenopus* ribosomal protein L5 to 5S rRNA. J Mol Biol. 2003; 330: 979–92 12860121 10.1016/s0022-2836(03)00685-5

[50] Szymanski M, Barciszewska MZ, Erdmann VA, Barciszewski J. 5 S rRNA: structure and interactions. Biochem J. 2003; 371: 641–51 doi: 10.1042/BJ20020872 12564956 10.1042/BJ20020872 PMC1223345

[51] Rideau AP, Gooding C, Simpson PJ, Monie TP, Lorenz M, Huttelmaier S, et al A peptide motif in Raver1 mediates splicing repression by interaction with the PTB RRM2 domain. Nat Struct Mol Biol. 2006; 13: 839–48 doi: 10.1038/nsmb1137 16936729 10.1038/nsmb1137

[52] Schellenberg MJ, Edwards RA, Ritchie DB, Kent OA, Golas MM, Stark H, et al Crystal structure of a core spliceosomal protein interface. Proc Natl Acad Sci U S A. 2006; 103: 1266–71 doi: 10.1073/pnas.0508048103 16432215 10.1073/pnas.0508048103 PMC1360545

