# Supplementary figures and images for: Non-canonical binding interactions of the RNA recognition motif (RRM) domains of P34 protein modulate binding within the 5S ribonucleoprotein particle (5S RNP)
Source: PLoS One. 2017 May 18;12(5):e0177890. doi: 10.1371/journal.pone.0177890 (PMC5436847; doi:10.1371/journal.pone.0177890)

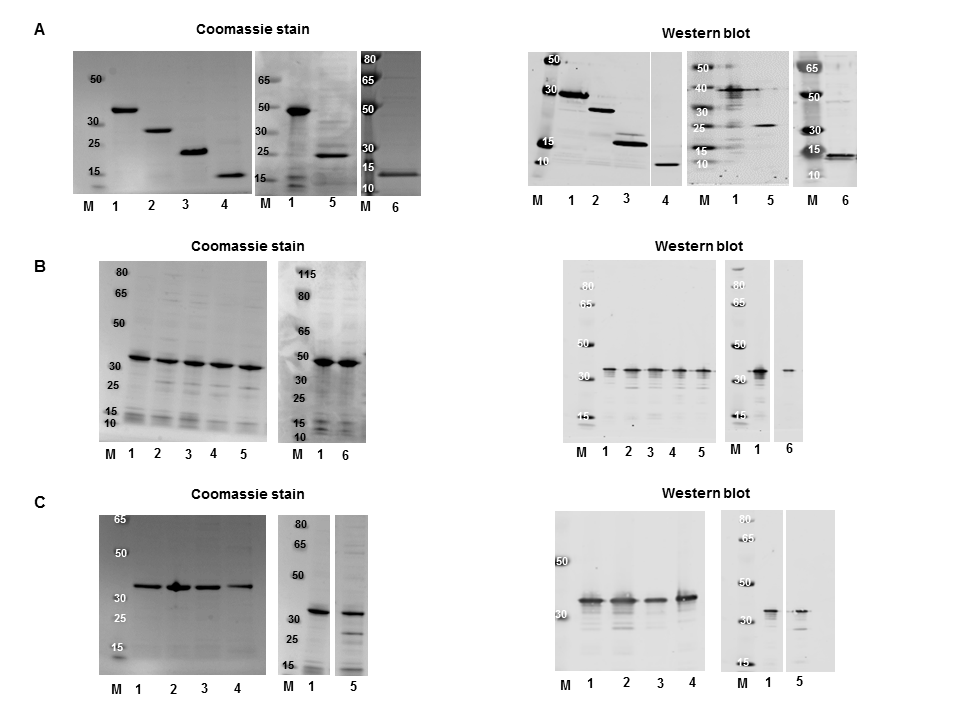

Supplement: S1 Fig — For the Commassie stain 10 μg of protein was analyzed and 1 μg of protein was analyzed for the western blot using anti–P34/P37 antibody. Panel A. Coomassie and western blot analysis of the P34 truncated proteins. Lane 1 = P34, lane 2 = N-R2, lane 3 = N-R1, lane 4 = R1 lane 5 = R1-R2, lane 6 = R2 (the difference in migration pattern is due to different gels (4–12% gradient or 10%) being used for protein analysis). The molecular weight for the P34 truncated proteins are as follows: P34 = 34 kDa, P34 N-R2 = 23 kDa, N-R1 = 14 kDa, R1 = 8.7 kDa. R1-R2 = 17 kDa, R2 = 8.8 kDa. Panel B. Coomassie and western blot analysis of P34 and P34 RNP mutated proteins. Lane 1 = P34, lane 2 = P34 Y60A, lane 3 = P34 Y92A, lane 4 = P34 F141A, lane 5 = P34 Y174A, lane 6 = P34 Y176A. The molecular weight for both P34 and the mutated P34 proteins is 34 kDa. Panel C: Coomassie and western blot analysis of P34 and P34 mutated proteins. Lane 1 = P34 protein, lane 2 = P34 R91A, lane 3 = P34 R91A, Y92A, lane 4 = P34 R88-91K, lane 5 = P34 R88-91A. The molecular weight for both P34 and the mutated P34 proteins is 34 kDa. (TIF) [file pone.0177890.s001.tif]

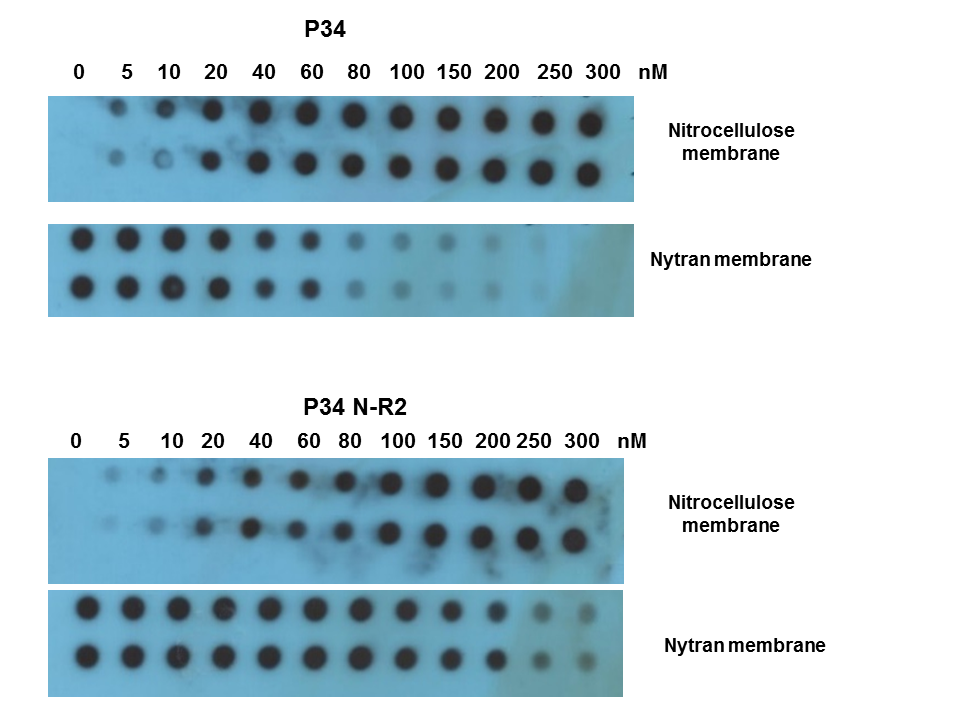

Supplement: S2 Fig — Nitrocellulose membranes were used to capture the protein-RNA complex and the nytran membranes were used to capture free unbound RNA. (TIF) [file pone.0177890.s002.tif]
